# Complex presentation of a left Fronto-zygomatic Dermoid cyst; a case report

**DOI:** 10.1093/jscr/rjae218

**Published:** 2024-04-10

**Authors:** Laith A Ayasa, Sara Rahhal, Alaa Khaled Najjar, Mohammed Aliwaiai, Asad Aldarawish, Izzeddin Bakri

**Affiliations:** Faculty of Medicine, Al Quds University, Jerusalem, Palestine; School of Medicine, The University of Jordan, Amman, Jordan; Department of Neurosurgery, Al-Makassed Islamic Charitable Hospital, Jerusalem, Palestine; Department of Neurosurgery, Al-Makassed Islamic Charitable Hospital, Jerusalem, Palestine; Department of Neurosurgery, Al-Makassed Islamic Charitable Hospital, Jerusalem, Palestine; Department of Pathology, Al-Makassed Islamic Charitable Hospital, Jerusalem, Palestine

**Keywords:** Dermoid cyst, Dermoid, Fronto-zygomatic

## Abstract

We present a case of craniofacial dermoid cyst in a 50-year-old female. The patient's complaint was persistent refractory headaches with no other significant neurological symptoms. Diagnostic imaging revealed the presence of a lesion in the left fronto-zygomatic region. Surgical intervention involved a craniotomy that led to a successful excision of the dermoid cyst. The diagnosis was subsequently confirmed by histopathological analysis. This case underscored the importance of considering DC as a potential diagnosis for any craniofacial lesion, given their diverse presentations and associated complications.

## Introduction

Dermoid cysts (DCs), recognized as benign, slowly proliferating congenital neoplasms, predominantly occur in children but can also emerge in adult patients [1]. DCs in the craniofacial region, particularly the fronto-zygomatic area, are rare and account for only a small percentage of all DCs [2]. The clinical manifestations of DCs in the craniofacial region may vary depending on the location and size of the cyst [3]. In this report, we present a case of a left fronto-zygomatic dermoid cyst in a 50-year-old female, detailing the clinical presentation, radiological findings, surgical management, and histopathological confirmation, thereby contributing to the existing literature on this rare and intriguing clinical entity.

## Case report

A 50-year-old female was admitted to our facility with a chronic history of cephalgia, characterized by gradual onset, progressive nature, and craniocaudal radiation. These headaches were refractory to standard analgesic therapy. The patient's medical history was notable for the absence of any seizure episodes or emesis.

**Figure 1:**
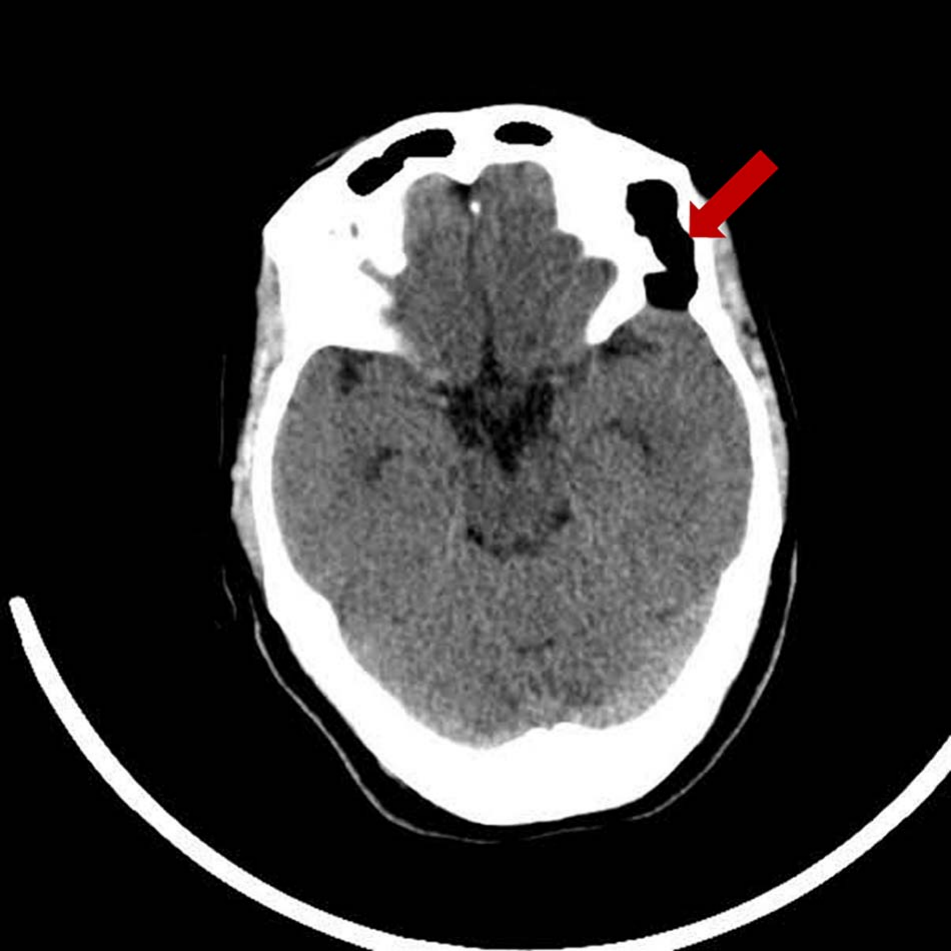
Pre-operative axial CT scan displaying a well-defined, low attenuating lesion in the left fronto-zygomatic region.

**Figure 2:**
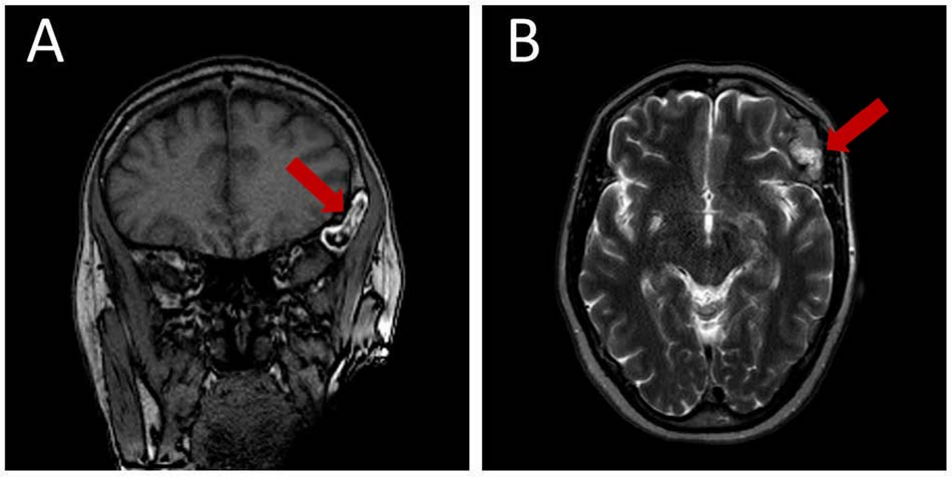
Pre-operative brain MRI exhibiting: (A) a high-intensity lesion in the left fronto-zygomatic region on coronal T1-weighted images (T1WI), and (B) Axial T2-weighted images (T2WI) depicting variable signal intensity ranging from iso- to hyperintense.

Prior diagnostic imaging included a computed tomography (CT) scan, revealing a hypodense lesion in the left fronto-zygomatic region (Fig. 1). Subsequent brain magnetic resonance imaging (MRI) showed a high-intensity lesion on T1-weighted images (T1WI) (Fig. 2A), and an iso-to-hyperintense lesion on T2-weighted images (T2WI) (Fig. 2B).

Upon admission, she was afebrile with stable vital signs; displaying a heart rate of 65/min, blood pressure of 130/70 mmHg, and a respiratory rate of 17/min. Neurological assessment revealed normal consciousness, orientation, and alertness, as evidenced by a Glasgow Coma Scale score of 15/15. The neurological examination was unremarkable, with intact cranial nerves, full limb movement, normal sensation, tone, reflexes, and the absence of Hoffman, Babinski, and Romberg signs.

The clinical course and the lesion necessitated the consideration of various intracranial lesions including epidermoids, teratomas, lipomas, craniopharyngiomas, and an infectious process. Craniopharyngiomas, often confused with dermoid cysts due to similar radiological features, predominantly occur in sellar and suprasellar regions, contrasting with our case's location. The possibility of an infectious process was negated by the absence of fever and normal inflammatory markers. Lipomas, although radiologically similar to dermoid cysts, consist solely of mature adipocytes, lacking the heterogeneity seen in dermoid cysts. Epidermoid cysts typically appear isointense on T1WI, in contrast to the hyperintense signal of dermoid cysts. Given these considerations, alongside the lesion’s hyperintense T1WI signal and its diverse tissue composition later confirmed by histopathology, a dermoid cyst was identified as the most probable diagnosis.

Following a comprehensive review of clinical and radiological features, and in consultation with the neurosurgery department, a dermoid cyst was diagnosed. The patient subsequently underwent a left fronto-orbito-zygomatic craniotomy extended to the orbital roof, involving excision of the bone tumor and reconstructive cranioplasty using bone cement. The mass and its capsule were successfully excised and submitted for histopathological analysis.

**Figure 3:**
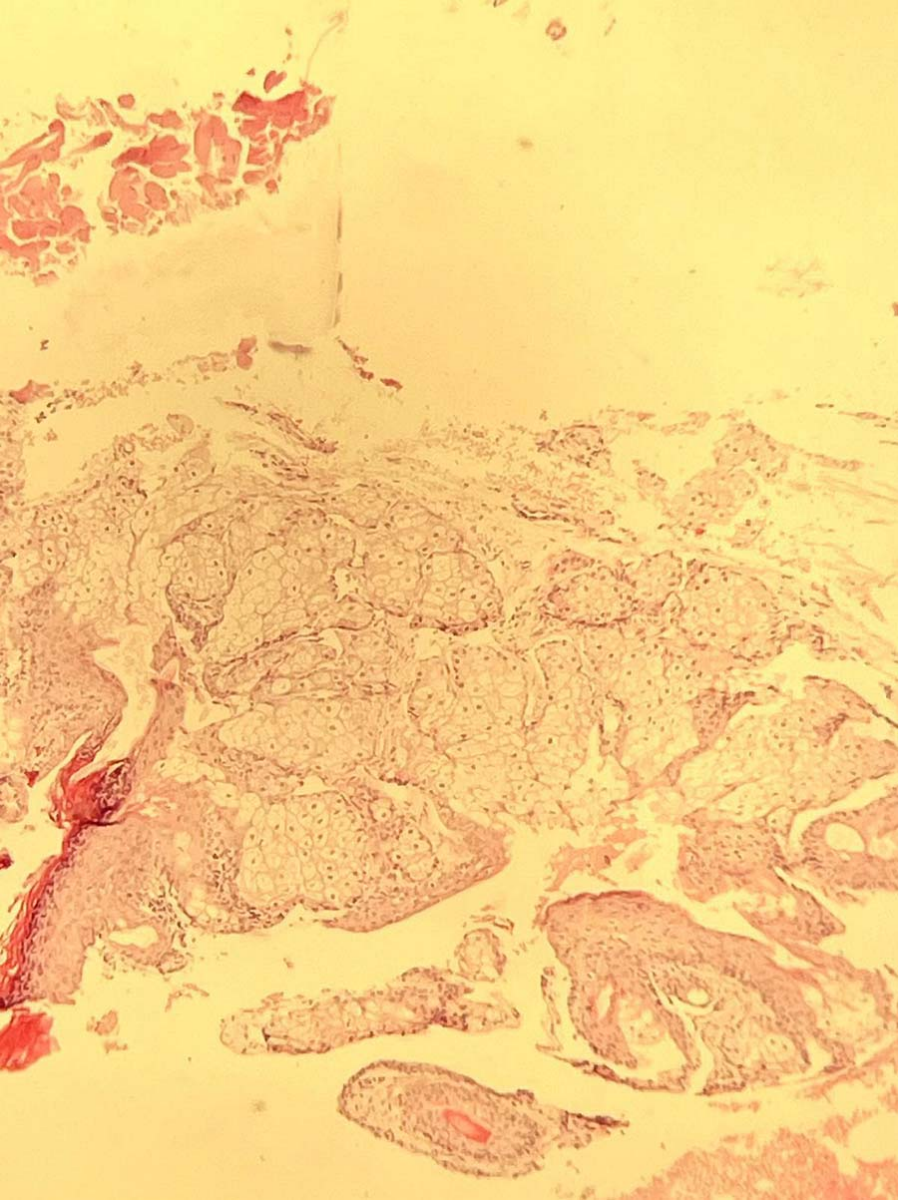
Histopathological slide showing a cystic structure lined by well-differentiated epidermis and dermis, filled with keratinous debris. [H&E stain, original magnification ×10].

The histopathological examination confirmed the presence of a dermoid cyst, characterized by stratified squamous epithelial lining, associated adipose tissue, keratinous debris, calcific depositions, and cholesterol pockets (Fig. 3).

The surgical procedure was executed without complications. Postoperatively, the patient was transferred to the Neurological Intensive Care Unit (ICU) and remained neurologically intact. Laboratory investigations during the hospital stay indicated normal serum electrolytes, renal function, and complete blood count parameters.

**Figure 4:**
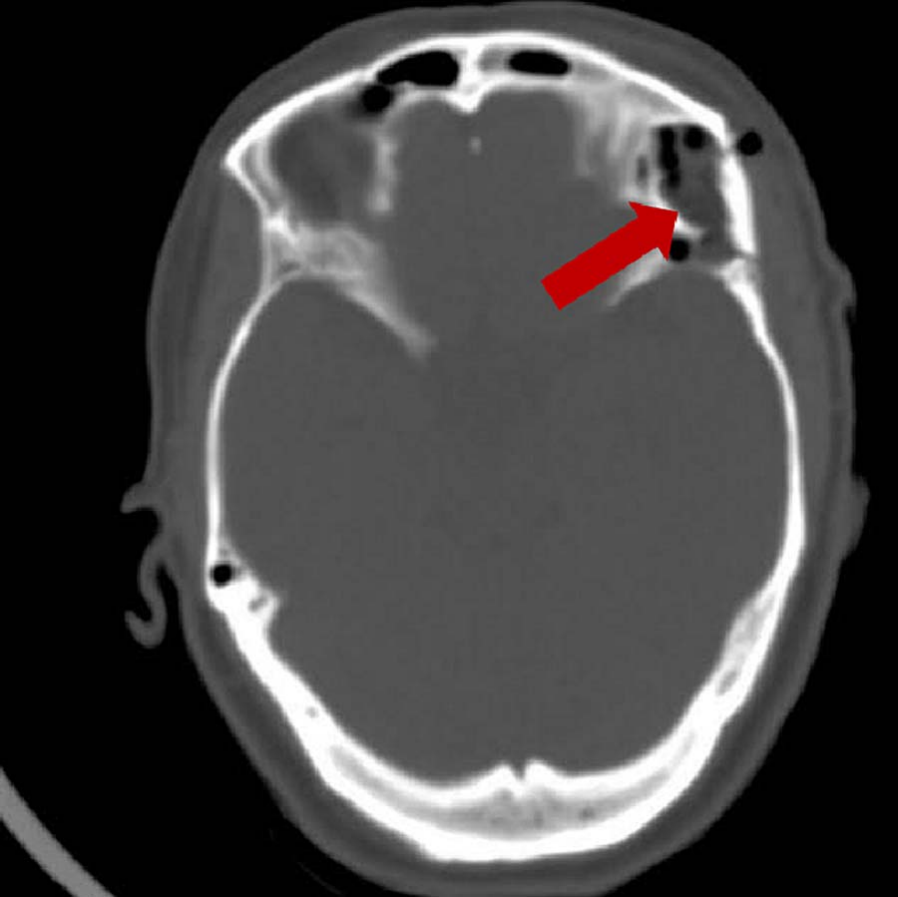
Post-operative brain axial CT scan illustrating the total resection of the lesion.

A post-operative brain CT scan demonstrated total resection of the lesion (Fig. 4). The postoperative course was uneventful, and the patient was discharged without any neurological deficits.

**Figure 5:**
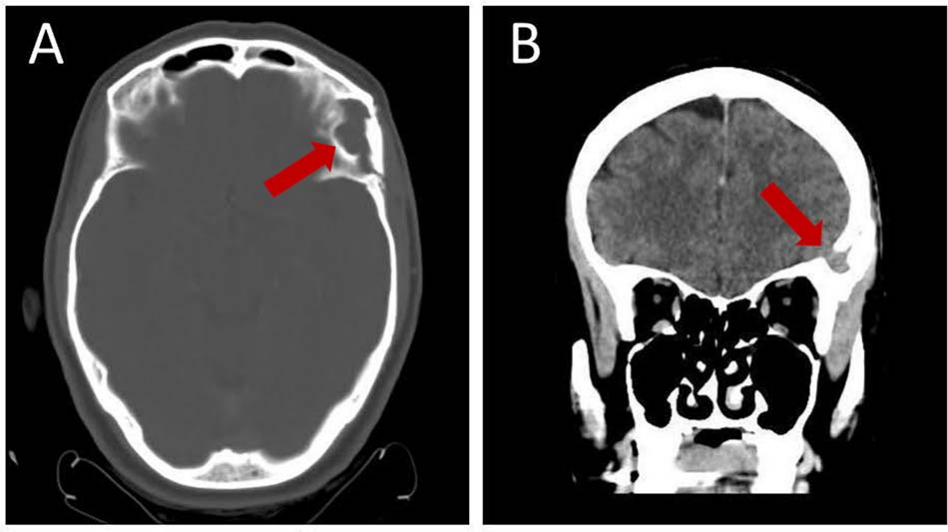
Follow-up brain CT, 3 months post-operation, showing complete resection of the lesion without residual tissue: (A) axial view and (B) coronal view.

A follow-up brain CT 3 months after the operation illustrates resection with complete absence of residual tissue (Fig. 5).

## Discussion

Dermoid cysts are benign lesions lined with keratinizing squamous epithelium, containing hair, sebaceous glands, sweat glands, and mesodermal derivatives. Adnexal tissue and mesodermal derivatives are pathognomonic for dermoid cysts rather than epidermoid cysts. Among dermoids, craniofacial DCs account for approximately 7% of cases, with an incidence ranging from 0.03 to 0.14%. The most common site for craniofacial DC is the periorbital region, specifically the fronto-zygomatic suture. However, they can also occur in other locations such as the nasal, intraoral, scalp, and postauricular regions. These cysts predominantly affect children under the age of 6, with most cases being identified before 3 months of age [10, 11]. However, our case is unique as our patient presented at 50 years of age. To contextualize our findings within the broader spectrum of craniofacial dermoid cyst presentations, diagnostics, and treatment outcomes, we have summarized and compared our case against similar reports in the literature in Table 1.

**Table 1:** Comparative analysis of similar cases in the literature.

**Author**	**Year published**	**Age**	**Sex**	**Lesion Site**	**Cyst size**	**Bilateral**	**Signs and Symptoms**	**Duration of symptoms**	**Operation**	**Outcome**	**Follow up**	**Recurrence**
Kumar *et al.,* 2017 [4]	2017	28	F	Right Superior orbital fissure	5.9 mm X 12.5 mm	No	Right eye ptosis and headache	1 month	TR	resolved	6 months	N/A
Howard *et al.,* 2019 [5]	2019	27	M	Left Frontozygomatic suture	2.0 X 2.1 X 1.1 cm	No	Diplopia, left sided headache, hypoglobus, proptosis	8 years, new onset headache	TR	resolved	N/A	N/A
Jorba *et al.,* 2019 [6]	2019	44	M	Left Frontozygomatic suture	N/A	No	Decreased visual acuity, pain, exophthalmos	3 weeks	TR	improved	6 months	No
Elahi and Glat, 2003 [7]	2003	4	F	lateral to The supraorbital rims	Left: 2 X 1.5 cm right: 1.3 X 1.1 cm	Yes	Asymptomatic, cosmetically concerning due to mild swelling	Progressive growing for 3.5 years	TR	resolved	2 years	No
Kang *et al.,* 2016 [8]	2016	13	M	superolateral to the orbital rim	Left: 1.2 cm X 0.7 cm Right: 1.5 cm X 0.9 cm	Yes	Asymptomatic, mild swelling	Progressive growing Since birth	TR	resolved	N/A	N/A
Samuelson *et al.,* 1988 [9]	1988	59	M	Left Frontozygomatic suture with temporal fossa extension	13 mm X 13 mm	No	Hypoglobus, diplopia	Few months	TR	resolved	N/A	N/A

Abbreviations: F = Female, M = male, N/A = not mentioned, TR = total resection

The majority of fronto-zygomatic DCs are asymptomatic but can present with visual and orbital manifestations, as well as aesthetic abnormalities [12]. In addition to our case, Howard et al. reported a 27-year-old patient presented with left hypoglobus, proptosis, diplopia, and a left-sided headache secondary to a DC located supraorbitally in the fronto-zygomatic suture [5]. While this case also included a headache, it differed from ours in terms of clinical presentation. To our knowledge, our case is the first reported instance of a fronto-zygomatic DC presenting solely with a headache, without any other symptoms. This further highlights the unique nature of our case. Additionally, it is important to note that headaches are more commonly reported in intracranial DCs and rarely observed in DCs found in the fronto-zygomatic region [13]. The presence of headaches in this case could be attributed to direct irritation of adjacent structures. Radiologically, the appearance of DCs depends on their content. CT scans can show hypodense regions due to fat and hair, and hyperdense regions due to calcification and hemorrhagic changes as well as any bony changes. On MRI, DCs appear hyperdense on T1-weighted and FLAIR sequences, and hypodense or heterogeneously hyperdense on T2-weighted sequences, which aligns with our case. The gold standard treatment for DC is complete surgical removal, with careful handling to prevent cyst rupture [14]. This unique case highlights the importance of considering DC as a potential diagnosis for any craniofacial lesion, given their diverse presentations and associated complications.
